# Selection of aptamers targeted to food‐borne pathogenic bacteria *Vibrio parahaemolyticus*

**DOI:** 10.1002/fsn3.1677

**Published:** 2020-06-03

**Authors:** Lan Wang, Shuxia Lyu, Ganyu Gu, Samantha Bolten

**Affiliations:** ^1^ College of Food Science and Engineering Shenyang Agricultural University Shenyang China; ^2^ College of Bioscience and Biotechnology Shenyang Agricultural University Shenyang China; ^3^ Environmental Microbiology and Food Safety Laboratory of USDA Agriculture Research Service at Beltsville Agriculture Research Center Beltsville Maryland USA

**Keywords:** NGS, qPCR; nano‐gold, Vibrio parahaemolyticus, X‐aptamer kit

## Abstract

*Vibrio parahaemolyticus* (*Vp*) is a common marine halophilic food‐borne pathogen, mainly found in seafood and food with a high salt content. Gastrointestinal reactions such as diarrhea, headache, vomiting, nausea, and abdominal cramps may occur after eating food infected with *Vp*. This study aimed to screen for high‐affinity aptamers that specifically recognize *Vp*. A high‐affinity modified aptamer screening kit was used to rapidly screen aptamers of the food‐borne *Vp*. The first round of screening involved release of target aptamers from the microspheres. The "false‐positive" aptamers were eliminated after specific binding to and elution of *Vp* in the second round. The second round of screening of the aptamers involved polymerase chain reaction (PCR), and the abundance of a sequence was determined using next‐generation sequencing. Nine high‐affinity aptamer sequences were obtained, and the first eight modified aptamer sequences were derived using a cloud‐based intelligent software of the American AM Biotech Co. *Escherichia coli (E. coli)* was used as a control, and aptamer ID 12 with the highest affinity for *Vp* was selected using real‐time PCR. According to the principle of color change caused by nano‐gold condensing under salt induction, *Salmonella*, *Listeria monocytogenes (L. monocytogenes)*, and *E. coli* were used as counter‐screening bacteria, and the aptamer ID12 was combined with nano‐gold. The results showed that aptamer ID12 has strong specificity for *Vp*. Based on these findings, this study developed a simple, innovative, and rapid method for screening *Vp* aptamers.

## Graphical abstract

Aptamers as novel biometric molecule recognition tools are being widely used for disease and food-borne pathogen detection, drug development, clinical diagnosis, and analytical chemistry. We used a kit specially designed by AM Biotech combined with qPCR and nano-gold to screen high-affinity, highly specific modified aptamers of Vp.

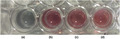

## 1 INTRODUCTION

*Vibrio parahaemolyticus* (*Vp*) is a common marine halophilic food‐borne pathogen, mainly found in seafood and food with a high salt content (De Paola, Nordstrom, & Bowers, 2003; Lozano León, Torres, Osorio Carlos, & Martínez Urtaza, 2003). Gastrointestinal reactions such as diarrhea, headache, vomiting, nausea, and abdominal cramps may occur after eating food infected with *Vp* (Liston, 1990; Yicheng & Chengchu, 2007). The data from several surveys show that food‐borne *Vp* infection has become a serious threat to public health worldwide (Nuo, 2013). In the coastal regions of developed countries such as the United States and Japan, multiple food‐borne diseases caused by *Vp* have been reported (Kiiyukia et al., 1989)*.* The methods for routine detection of *Vp* mainly include microbial testing techniques, instrumental analysis methods such as real‐time polymerase chain reaction (PCR) (Luyan, Cai, & Jingdong, 2006; Zhongmin, Ming, & Yongfen, 2007), molecular biology techniques, and immunological detection methods such as enzyme‐linked immunosorbent assay (ELISA) (Hochel, Viochna, Skvor, & Musil, 2004), enzyme‐linked fluorescence analysis (ELFA) (Shigeko & Yoshihiro, 2010), time‐resolved fluorescence immunoassay (TrFIA) (Sinikka, Harri, & Mika, 2007), and chemiluminescence immunoassay (CIA) (Mathew, Alagesan, & Alocilja, 2004). However, these methods have some specific limitations/disadvantages for *Vp* detection. For example, the traditional microbial testing techniques are time‐consuming, require complicated operation, and frequently have a low diagnostic sensitivity and specificity; the PCR and immunological detection methods are limited by a high false‐positive rate, high cost, and lack of stability. Therefore, methods for rapid detection of food‐borne *Vp* are urgently required.

Aptamers, as a new type of biosensor, were developed almost a decade ago. Aptamers are usually single‐stranded 10–100 nucleotide‐long DNA or RNA molecules that are amplified and screened using SELEX (Systematic Evolution of Ligands by Exponential Enrichment) (Duan, Wu, Chen, Huang, & Wang, 2012). In the 1990s, Ellington and Szostak (Mairal, Ozalp, Sanchez, & Mir, 2008) and Tuerk and Gold (Jenison, Gill, Pardi, & Polisky, 1994) screened RNA ligands that showed high affinity and specific binding to T4 DNA polymerase using SELEX and named them aptamers, derived from the Greek word “aptus” (meaning “to fit”) Bunka and Stockley (2006).

Aptamers represent a novel and highly stable biometric molecule recognition tool that can specifically bind to proteins or other small molecules and are easy to modify Junli et al., (2015). They have a broad range of targets, including simple organic and inorganic small molecules, peptides, proteins, and even virus particles, bacteria, eukaryotic cells, and tissues (Shamar, Helly, & Cload, 2008). In the presence of the target, an aptamer can fold to form a highly ordered three‐dimensional structure and can bind to its target via hydrogen bonds, hydrophobic interactions, van der Waals forces, and/or other noncovalent interactions with high specificity and affinity (Hwang, Ko, Lee, & Kang, 2010; Levy‐Nissenbaum, Radovic‐Moreno, & Wang, 2008; Liu, Shi, Chen, & Duan, 2014; Sefah, Tang, Shangguan, & Chen, 2009; Shangguan, Meng, Cao, & Xiao, 2008). The target detection ability of aptamers depends on the affinity and specificity of the antibody, which overcomes many shortcomings of the conventional detection methods mentioned above (Nuo, 2013). Since their development, aptamers have been widely used in various areas (Eaton, 1997), such as in disease identification, medicine development, clinical diagnosis, analytical chemistry, and food‐borne pathogen detection.

The SELEX process commonly applied for aptamer screening is time‐consuming and cumbersome, mainly due to the large number of screening rounds and the adverse effects of external factors during processing, such as the washing solution used for elution. To the best of our knowledge, the present study is the first to use the X‐aptamer kit for aptamer screening of the food‐borne pathogenic bacteria *Vp*. Potential aptamers were sequenced, modified, and confirmed to be potentially useful for *Vp* detection and further research.

Gold nanoparticles/nano‐gold (AuNPs) are generally prepared by reducing chloroauric acid by trisodium citrate; the resulting solution is a colloid, the stability of which is maintained by electrostatic repulsion between the negatively charged citrate‐coated AuNP particles. Addition of high concentration of NaCl generally destroys the stability of the colloidal solutions, leading to aggregation, which is observed as a change in the color of the colloidal solution from red to blue (Li & Rothberg, 2004; Zhao, Chiuman, & Lam, 2008). Unlike double‐stranded (ds)DNA, the positively charged bases in single‐stranded (ss)DNA are exposed in the free state, which enables their direct adsorption onto the negatively charged nano‐gold surface via electrostatic interactions. When the nano‐gold adsorbed with the aptamer remains stable under a high concentration of salt solution, the solution stays red, and the solution will turn blue again when a target is present in the solution. The present study used this principle of color response to specifically screen aptamers for *Vp.*

## 2 MATERIALS AND METHODS

### 2.1 Bacterial strains and preparation

The following bacterial strains were used in this study: *V. parahaemolyticus* ATCC 17802, *Staphylococcus aureus* (*S. aureus*) ATCC 6538, *Escherichia coli* (*E. coli*) ATCC 25922, *Listeria monocytogenes (L. monocytogenes)* FSCC 178006, and *Salmonella* ATCC 14028. *Vp* was used as the target and was grown overnight at 37℃ in alkaline peptone water (APW; Hopebio, Qingdao, China) culture medium (10 g peptone and 30 g NaCl/L, pH 8.5 ± 0.2). As screen controls, *Salmonella, L. monocytogenes, and E. coli* were grown overnight at 30°C in brain heart infusion (BHI; Hopebio). *S. aureus*, another screen control, was grown in Luria Bertani culture media (LB; Land Bridge Technology). Bacterial cells were harvested while in log phase of growth. The experimental reagents chloroauric acid (HAuCl_4_.4H_2_O, Shanghai McLean Biochemical Technology Co., Ltd.) and trisodium citrate (Na_3_C_6_H_5_O_7_.2H_2_O, Shenyang No.1 Reagent Factory) were analytical grade.

### 2.2 Aptamer screening using the X‐aptamer selection kit

Aptamers targeting *Vp* were screened using an X‐aptamer selection kit (American AM Biotech Co., Ltd.), containing a 10^9^‐microsphere library, as well as forward and reverse primers, per the manufacturer's instructions. Bovine serum albumin (BSA) and all PCR reagents were purchased from Shanghai Sangon Biological Science & Technology Company. All PCR amplifications were performed using a S1000 thermal cycler (Bio‐Rad Laboratories [Shanghai] Co., Ltd.). A JW‐2017HR high‐speed refrigerated centrifuge was used for centrifugation during all purification steps. The steps of aptamer screening are described below.

#### 2.2.1 Preparation of oligonucleotide library

The dry microsphere library was completely wet‐transferred using buffer B (1 × phosphate‐buffered saline [PBS], pH 7.4, 0.02% Tween 20, 1 mM MgCl_2_) to centrifuge tubes (5 times, 2 ml each time). The tubes were centrifuged at 3,000 rpm for 10 min at room temperature. The supernatant was carefully discarded, and only 100 μl of the supernatant was retained in the tube. Buffer B was added to the above centrifuge tube to make up the volume to 500 μl. Then, 500 μl of 1 N NaOH was added and incubated for 30 min in a 65 ℃ water bath. The reaction was neutralized by the addition of 400 μl of 2 M Tris‐HCl. Ten fresh filter columns were prepared according to the operating manual.

#### 2.2.2 Negative screening

*Staphylococcus aureus* was cultured overnight, and approximately 10^7^ cells were collected via centrifugation. The cells were then washed thrice with buffer A (1 × PBS, pH 7.4, 0.02% Tween 20, 1 mM MgCl_2_, 2 mg/ml BSA). The supernatant was discarded, followed by the addition of the prepared aptamer microsphere library and incubation at room temperature with gentle rotation. After 1 hr, the mixture was centrifuged at 3,000 rpm for 5 min, and the precipitate was retained and washed thrice with buffer A by centrifugation at 1,500 rpm for 10 min. The supernatant from each wash was retained, and all supernatants were combined for positive selection (positive screening library). The above precipitate was resuspended in 50 μl buffer A and denatured at 95 ℃ for 10 min. Subsequently, the suspension was centrifuged at 12,000 rpm for 5 min, and the supernatant was collected for use in the negative screening in the second pull‐down screen.

#### 2.2.3 Positive screening

*Vp* was cultured overnight. Approximately 10^7^ cells were collected via centrifugation, and the cells were washed thrice with buffer A, followed by addition to the positive screening library retained during the negative screening and incubated at room temperature for 1 hr. Next, the cells were centrifuged at 3,000 rpm for 5 min and washed thrice with buffer A, each time at 1,500 rpm for 10 min, and the supernatant was discarded. One hundred microliters of buffer A was added to the precipitate and denatured at 95°C for 10 min. This was followed by centrifugation for 5 min at 12,000 rpm to collect the supernatant, which was used for the positive screening in the second pulldown screening.

#### 2.2.4 Second pulldown screening

**Figure 1:**
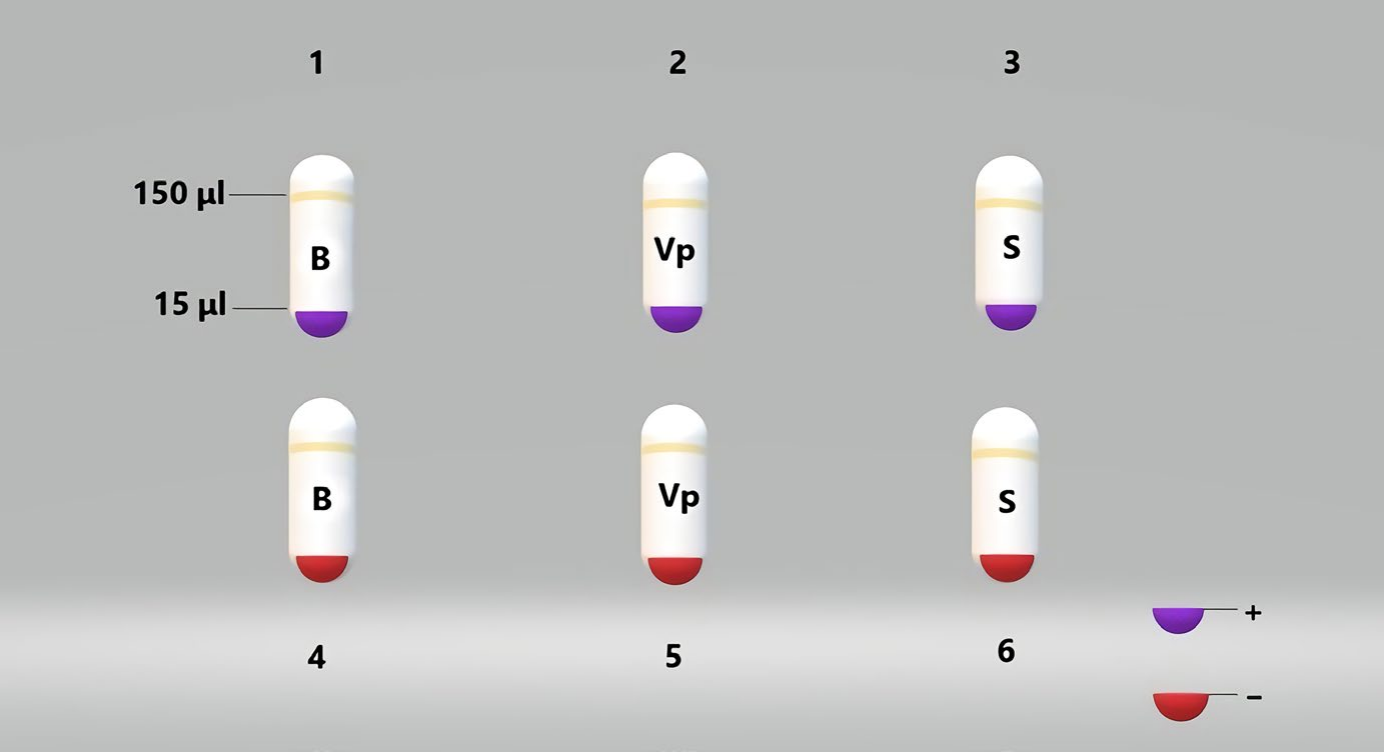
Samples of the second pulldown screening. B: buffer B; *Vp*: *Vibrio parahaemolyticus*; S: *S. aureus*; +: positive; ‐: negative

The total reaction volume per tube was 150 μL. Forty‐five microliters of the lysed oligonucleotide pool from the positive selection was aliquoted into three tubes (labeled 13); Similarly, 45 μL of the lysed oligonucleotide pool from the negative selection was aliquoted into three tubes (labeled 4–6) (Figure 1). One hundred thirty‐five microliters of buffer B was added to tubes #1 and #4, while 135 μL buffer B containing *Vp* was added to tubes #2 and #5. One hundred thirty‐five microliters of buffer B containing *S. aureus* was added to tubes #3 and #6. All tubes were incubated for 30 min at room temperature. The cells in tubes #2, 3, 5, and 6 were recovered by centrifugation. The precipitate was washed once with buffer solution A and resuspended in 100 μL buffer A (2 μL was used as a template for PCR).

#### 2.2.5 PCR and gel electrophoresis

PCR amplification was performed using each of the above reactions in 20 μL total volume, which contained 1 × Go Taq® Buffer, 0.2 mM dNTP, 2.5 mM MgCl_2_, 1 U Taq polymerase, 0.4 μM forward constant region primer, and 0.4 μM reverse constant region primer. The amplification conditions were as follows: predenaturation at 94 ℃ for 1 min, cycling (20 times) at 94 ℃ for 30 s, 50 ℃ for 30 s, and 72 ℃ for 1 min, and final extension at 72 ℃ for 3 min. Agarose gel electrophoresis was performed on 4.5% agarose gel at 200V for 30 min.

### 2.3 Next‐generation sequencing

The sequences of the potential isolated aptamers were identified via next‐generation sequencing of 10 ng of each PCR product in the Next‐generation Sequencing Department of the Shanghai Sangon Biological Science & Technology Company. Sequencing data were analyzed by the AM Corporation. After data analysis, oligonucleotides with high frequencies were further modified and synthesized for subsequent confirmation tests. Synthesis was performed on the 50 nmol scale without any purification.

### 2.4 Binding affinity of candidate aptamers to Vp using qPCR

To confirm the synthesized aptamers, binding affinity and specificity experiments were performed using qPCR. The *Vp* strain ATCC 17,802 was used for affinity studies. An exclusivity assay was performed using *E. coli* ATCC 25,922. In all cases, bacterial cells were grown overnight (10^7^–0^8^ CFU/ml) and then incubated with 100 nM individual aptamer candidates for 1.5 hr at room temperature. After centrifugation at 3,000 rpm for 3 min, the cells were washed thrice and resuspended in 300 μl buffer B. Each group was denatured at 100°C for 10 min and centrifuged at 10,000 rpm for 5 min, and the supernatant was collected as the template for qPCR tests.

The aptamers were 10‐fold serial diluted to 10 nM, 1 nM, 0.1 nM, 0.01 nM, and 0.001 nM for preparation of the standard curve. Two‐microliter aliquots of the aptamer solution were mixed with 10 μL GoTaq® qPCR master mix (2×), 0.4 μl 10 μM forward primer, 0.4 μl 10 μM reverse primer, and 7.2 μL nuclease‐free water in a total volume of 20 μL. qPCR was performed in a 96‐well PCR plate (Sarstedt) covered with strip caps. The thermal cycling regimen was as follows: initial denaturation for 3 min at 95°C, cycling (45 times) for 30 s at 95°C, 30 s at 57°C, and 30 s at 72°C on the ABI 7,500 real‐time PCR machine (Thermo Fisher Scientific).

### 2.5 Binding specificity of candidate aptamers to Vp using nano‐gold

#### 2.5.1 Preparation and characterization of nano‐gold

Nano‐gold was prepared by the trisodium citrate reduction method (Cheung et al., 2013). First, we prepared 1% chloroauric acid solution and 1% trisodium citrate solution for later use. Then, 4.2 ml of chloroauric acid solution was added to 95.8 ml of ultrapure water for continuous boiling for 10 min before rapid addition of 10 ml trisodium citrate solution. After the color of the solution turned red within 1 min, the heating was stopped, and the solution was left to cool to room temperature. The wine‐red liquid obtained was nano‐gold. The size and morphology of the prepared nano‐gold particles were characterized by transmission electron microscopy (TEM). For TEM analysis, 5 µl of the prepared nano‐gold was transferred onto a carbon‐coated copper mesh, placed it an electric blast drying oven at 60°C for 1 hr, and then placed in the TEM sample stage. TEM images were taken at an operating voltage of 80.0 kV.

#### 2.5.2 Specificity test

Using the same concentration of *L. monocytogenes*, *Salmonella*, and *E. coli* as interfering bacteria, with the same concentrations of aptamer ID12, nano‐gold, and salt, the color change of the solution was monitored to observe the specificity of aptamer ID12.

## 3 RESULTS

### 3.1 PCR‐mediated detection of potential Vp aptamers screened using the X‐aptamer kit

**Figure 2:**
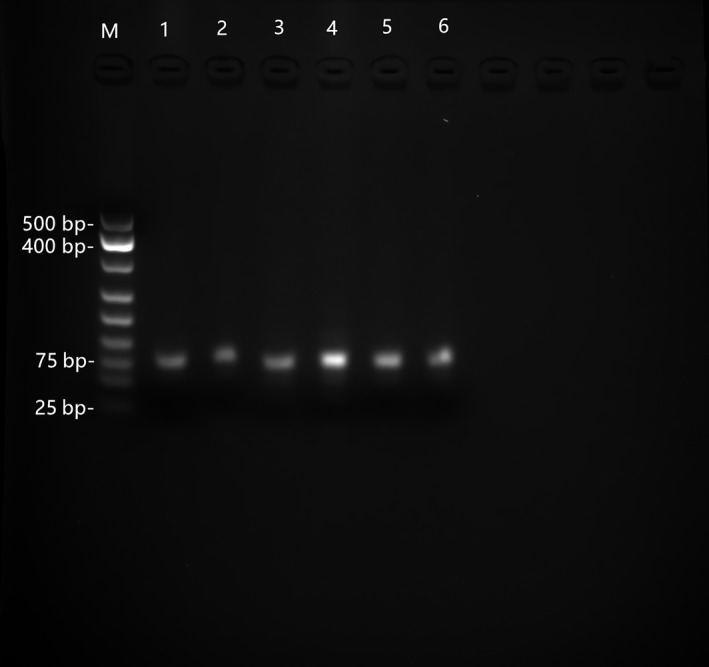
Results of gel electrophoresis of ssDNA products generated using 20 PCR cycles. M: DNA marker; 1: positive initial solution control; 2: positive selection pool of *Vp*; 3: positive selection pool of *S. aureus*; 4: negative initial solution control; 5: negative selection pool of *Vp*; 6: negative selection pool of *S. aureus*

As can be seen in Figure 2, the expected PCR band size was 75 bp. The number of cycles to be used for amplifying the target pool was important, as nonspecific products might appear with an increase in the number of cycles. PCR amplification was performed at 4 cycle intervals in 20 μl reactions per tube, and the number of cycles required to generate abundant and clean bands in agarose gel electrophoresis was used to determine the optimal number of PCR amplification cycles. The results of agarose gel electrophoresis indicated that a single band of the correct size was obtained after 20 cycles. Thus, 20 cycles were selected as the standard in this study.

### 3.2 Next‐generation sequencing of the isolated aptamers

**Figure 3:**
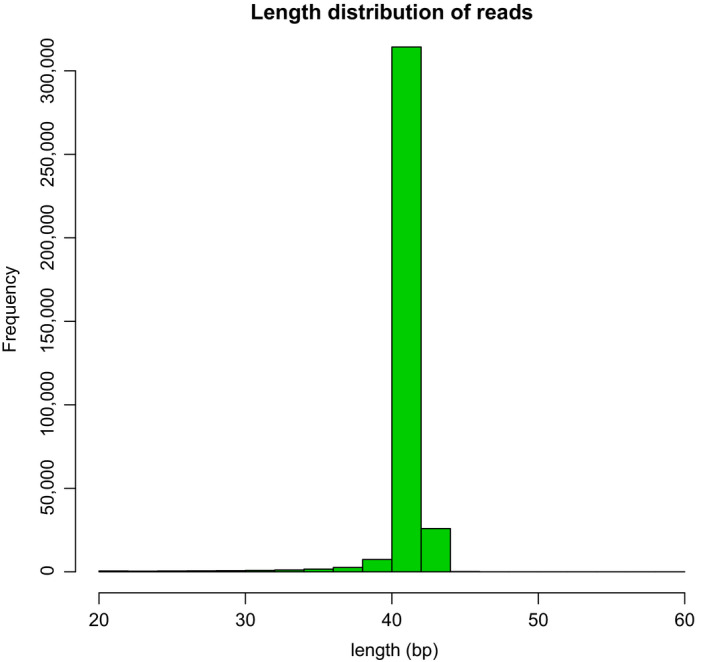
Nucleic acid aptamer sequence length distribution

In total, 543,367 reads were sequenced using Illumina MiSeq in the range of 20–56 bp. Forty‐two base pair‐long sequences were the most dominant fragment with 437,872 reads (Figure 3).

Sequence counts were normalized across all fractions used in the second selection stage (solution pull down). Sequences for each target were selected based on enrichment over normalized counts for: (a) the starting pool and (b) the magnetic particle only control. Enrichment of individual sequences was good for this selection. These proprietary methods resulted in the ranking of nine X‐aptamer candidates listed in Table 1. Sequence ID was coded to identify the fraction and/or target.

**Table 1:** X‐Aptamer candidates

ID	Name	Sequence
5	S.184004.T1.01	GCCCACAGACGTTCGGCAGGCACAGTTTGTCAAGGTCGTG
6	S.184004.T1.02	GCCCACCTCGCTGTGCAAGGCGAACGCCATCAGTGTGGGC
7	S.184004.T1.03	GCCCACCTCCGTTCGAAGGCCACAGCTCATGCGCGTGGGC
8	S.184004.T1.04	CACGACCCCACTGTGCGAGCCGAACACCACCACGGTGGGC
9	S.184004.T1.05	CACGACTTAGCYGTGTGGGCCGAACACCAGGCACGTGGGC
12	S.184004.T1.06	CACGACAGCAGTTCGGCGGGCACAGTGCGTGCGAGTCGTG
13	S.184004.T1.07	CACGACCATACTGTGGCAGACGAACGCCGTCACAGTGGGC
14	S.184004.T1.08	GCCCACGCGGCTGTGAGTCGCACAGCCATAGCACGTGGGC
15	S.184004.T1.09	GCCCACTGAACTGTGGCGGGCACAGGATGTGGAAGTGGGC

Nine unmodified aptamers were predicted using the quadruplex forming G‐rich sequences (QGRS, http://bioinformatics.ramapo.edu/QGRS/index.php) software and are listed in Table 2. The aptamer ID15 was calculated to form a G‐quadruplex with a score of 21, indicating that probability of the aptamer ID15 to form a ssDNA G‐quadruplex structure was high.

**Table 2:** Results of QGRS prediction

Position	Length	QGRS	G‐score
21	20	GGCACAGGATGTGGAAGTGG	21

Note

Underline letters (GG) means Min G‐Group Size is 2.

To compare the affinity of the modified aptamer and the unmodified aptamer, the first eight aptamers were modified. The modified aptamer sequences are shown in Table 3. The letters W, Y, and X denote the modified nucleotides indole‐dU, phenol‐dU, and amine‐dU, respectively. X‐aptamer candidates were resynthesized as oligonucleotides consisting of a 42‐bp random region, five additional Ts at the 5′‐end, and CCATG at the 3′‐end.

**Table 3:** Sequences identified for synthesis by AM Biotech

ID	Name	Sequence with modifications
5	S.184004.T1.01	TTTTTAAGCCCACAGACGWYCGGCAGGCACAGTYYGTCAAGGXCGYGCCATG
6	S.184004.T1.02	TTTTTAAGCCCACCYCGCYGTGCAAGGCGAACGCCATCAGTGTGGGCCCATG
7	S.184004.T1.03	TTTTTAAGCCCACCYCCGWYCGAAGGCCACAGCYCATGCGCGTGGGCCCATG
8	S.184004.T1.04	TTTTTAACACGACCCCACYGTGCGAGCCGAACACCACCACGGTGGGCCCATG
9	S.184004.T1.05	TTTTTAACACGACXYAGCYGTGWGGGCCGAACACCAGGCACGTGGGCCCATG
12	S.184004.T1.06	TTTTTAACACGACAGCAGWYCGGCGGGCACAGTGCGTGCGAGXCGYGCCATG
13	S.184004.T1.07	TTTTTAACACGACCAWACYGTGGCAGACGAACGCCGTCACAGTGGGCCCATG
14	S.184004.T1.08	TTTTTAAGCCCACGCGGCYGTGAGXCGCACAGCCAWAGCACGTGGGCCCATG

### 3.3 Binding affinity tests

Nine candidate aptamers were incubated with *Vp* and *E. coli*, and the positive screening process was repeated. The obtained supernatant was subjected to qPCR, and the values obtained are shown in Table 4. The highest ratio in Table 4 shows that the No. 6 (ID 12) candidate aptamer possessed the highest affinity.

**Table 4:** Results of qPCR

Well name	Ct	Quantity	Ratio
V1	20.7426	0.05508	1,343.41
E1	32.87716	4.1E−05	
V2	32.10162	——	
E2	32.10314	——	
V3	14.84586	1.256966	855.07
E3	29.11471	0.00147	
V4	18.29653	0.052801	1,200.02
E4	30.51482	4.45E−05	
V5	22.79037	0.368701	0.02
E5	14.25837	15.59137	
V6	19.26015	0.114346	1815.015
E6	31.74648	6.32E−05	
V7	20.31481	0.090709	890.73
E7	31.98106	0.000109	
V8	37.23086	0.015177	2.29
E8	39.33905	0.006629	
V9	12.74703	0.028146	382.42
E9	30.40216	7.37E−05	

Note

V: *Vp*; E: *E. coli*; 1–8: modified X‐Aptamer candidates; 9: unmodified X‐Aptamer candidate; Ratio: value of the quantity V to quantity E

### 3.4 Characterization of nano‐gold

**Figure 4:**
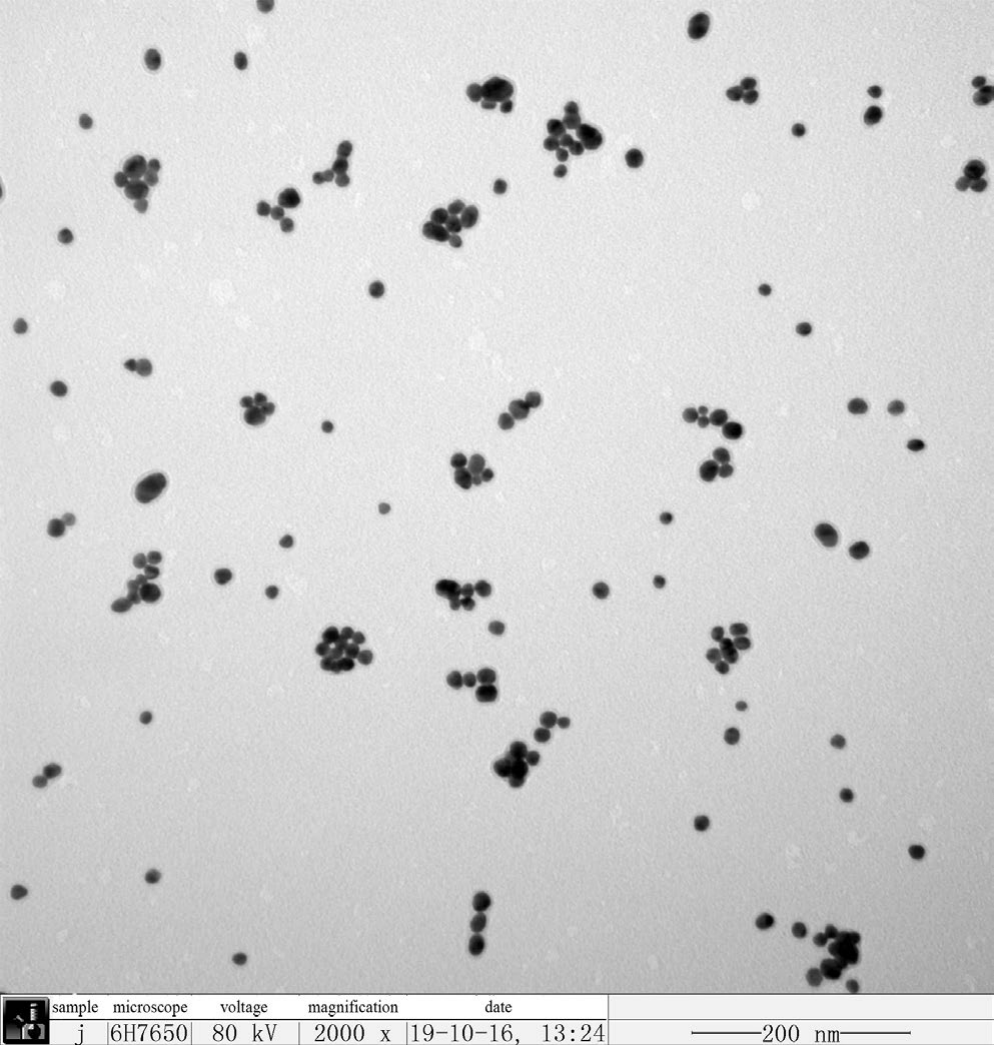
TEM image of nano‐gold

**Figure 5:**
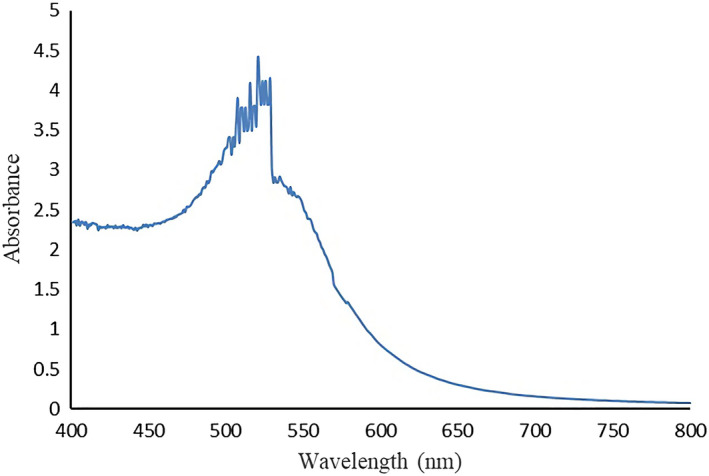
UV‐Vis absorption spectrum of nano‐gold

The TEM characterization results of nano‐gold prepared by trisodium citrate are shown in Figure 4. The diameter of the nano‐gold particles ranged from 13 to 17 nm, and the particles were dispersed and uniform in size. Because nano‐gold has unique light absorption characteristics, it is visible in the ultraviolet spectrum. There is a maximum absorption peak at 520 nm in the absorption spectrum (Figure 5).

### 3.5 Binding specificity tests

**Figure 6:**
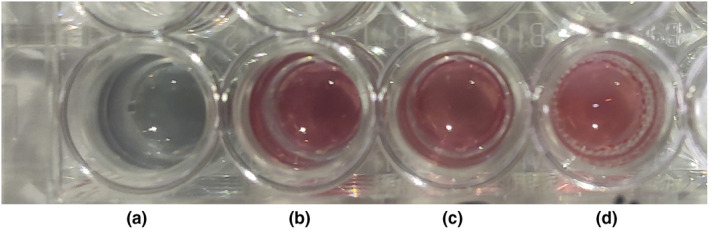
Specificity assay of *Vp* aptamer by color response of nano‐gold

As shown in Figure 6, after the target bacteria *Vp* was added to the a‐well, the color of the solution changed from red to blue, which conformed to the detection principle described in the Introduction. To ensure the consistency of aptamer, nano‐gold, and salt, *L. monocytogenes*, *Salmonella*, and *E. coli* were added to wells b, c, and d, respectively, with the same bacterial solution concentration. No aggregation or color change was observed in wells b, c, and d. Thus, aptamer ID 12 showed specific binding to *Vp*.

## 4 DISCUSSION

In this study, we used the kit specially designed by AM Biotech Co. to screen high‐affinity modified aptamers of *Vp*. In the first round of screening, the enriched target aptamers were released from the microspheres, then specifically bound to the target in the second round, and were subsequently eluted to exclude "false positive" aptamers. The candidate aptamers were amplified using PCR, and the sequence and abundance of the aptamers were obtained via next‐generation sequencing. The modified aptamer sequences were derived and synthesized by the American AM Biotech Co. High‐affinity sequences were determined using real‐time PCR. Two types of counter‐bacteria were selected, *S. aureus* which is different in morphology from *Vp*, and *E. coli* which is similar in morphology to *Vp*. The aim was to screen for the most compatible affinity among *Vp* aptamers. When using the nano‐gold color reaction principle to detect the specificity of aptamer ID 12, the presence of salt caused the nano‐gold solution to agglomerate. *Vp* is a halophile, and so there will be salt in the medium. In the specificity test, the target *Vp* should be removed from the culture medium to remove excess salt. The results showed that aptamer ID 12 specifically bound to *Vp* with high affinity. Compared to the traditional screening method, SELEX, the AM X‐aptamer screening kit offers a considerable reduction in the screening time (8–10 rounds for SELEX), and X‐aptamer libraries can accommodate different modifications, such as multiple modified versions of Lokesh, Wang, Lam, Thiviyanathan, and Ward (2017) as described in this study. The results of qPCR showed that the modified aptamer has higher affinity than the unmodified aptamer. The application of nanogold to the screening of aptamers can produce more intuitive results, and the specificity of the aptamers is visible to the naked eye. The importance of this study is that the selected optimal aptamer can be combined with nanoparticles to construct a series of new methods and techniques, such as quantitative detection of *Vp*, to provide more efficient, accurate, sensitive, and rapid detection of food‐borne pathogenic bacteria such as *Vp*. The results provide critical insight for improving food safety testing technology and can significantly promote the scientific utility of aptamers. At the same time, these findings provide innovative ideas for new methods to advance food safety testing technology.

## CONFLICT OF INTEREST

To the best of our knowledge, the named authors have no conflict of interest, financial, or otherwise.
